# Health, Lifestyle, and Psycho-Social Determinants of Poor Sleep Quality During the Early Phase of the COVID-19 Pandemic: A Focus on UK Older Adults Deemed Clinically Extremely Vulnerable

**DOI:** 10.3389/fpubh.2021.753964

**Published:** 2021-10-28

**Authors:** Chinedu T. Udeh-Momoh, Tamlyn Watermeyer, Shireen Sindi, Parthenia Giannakopoulou, Catherine E. Robb, Sara Ahmadi-Abhari, Bang Zheng, Amina Waheed, James McKeand, David Salman, Thomas Beaney, Celeste A. de Jager Loots, Geraint Price, Christina Atchison, Josip Car, Azeem Majeed, Alison. H. McGregor, Miia Kivipelto, Helen Ward, Lefkos T. Middleton

**Affiliations:** ^1^Ageing Epidemiology Research Unit (AGE), School of Public Health, Faculty of Medicine, Imperial College London, London, United Kingdom; ^2^Department of Psychology, Faculty of Health and Life Sciences, Northumbria University, Newcastle upon Tyne, United Kingdom; ^3^Edinburgh Dementia Prevention, Centre for Clinical Brain Sciences, University of Edinburgh, Edinburgh, United Kingdom; ^4^Division of Clinical Geriatrics, Center for Alzheimer Research, Karolinska Institutet and Karolinska University Hospital, Stockholm, Sweden; ^5^MSk Lab, Faculty of Medicine, Imperial College London, London, United Kingdom; ^6^Department of Primary Care and Public Health, Imperial College London, London, United Kingdom; ^7^Public Health Directorate, Imperial College Healthcare NHS Trust, London, United Kingdom; ^8^Institute of Public Health and Clinical Nutrition and Institute of Clinical Medicine, Neurology, University of Eastern Finland, Kuopio, Finland; ^9^Centre for Population Health Sciences, Lee Kong Chian School of Medicine, Nanyang Technological University, Singapore, Singapore; ^10^Department of Surgery and Cancer, Imperial College London, London, United Kingdom; ^11^Theme Aging, Karolinska University Hospital, Stockholm, Sweden; ^12^Patient Experience Research Centre, School of Public Health, Faculty of Medicine, Imperial College London, London, United Kingdom

**Keywords:** sleep quality, COVID-19 lockdown, clinically extremely vulnerable older adults, modifiable factors, sex differences

## Abstract

**Background:** Several studies have assessed the impact of COVID-19-related lockdowns on sleep quality across global populations. However, no study to date has specifically assessed *at-risk* populations, particularly those at highest risk of complications from coronavirus infection deemed “clinically-extremely-vulnerable-(COVID-19CEV)” (as defined by Public Health England).

**Methods:** In this cross-sectional study, we surveyed 5,558 adults aged ≥50 years (of whom 523 met criteria for COVID-19CEV) during the first pandemic wave that resulted in a nationwide-lockdown (April–June 2020) with assessments of sleep quality (an adapted sleep scale that captured multiple sleep indices before and during the lockdown), health/medical, lifestyle, psychosocial and socio-demographic factors. We examined associations between these variables and sleep quality; and explored interactions of COVID-19CEV status with significant predictors of poor sleep, to identify potential moderating factors.

**Results:** Thirty-seven percent of participants reported poor sleep quality which was associated with younger age, female sex and multimorbidity. Significant associations with poor sleep included health/medical factors: COVID-19CEV status, higher BMI, arthritis, pulmonary disease, and mental health disorders; and the following lifestyle and psychosocial factors: living alone, higher alcohol consumption, an unhealthy diet and higher depressive and anxiety symptoms. Moderators of the negative relationship between COVID-19CEV status and good sleep quality were marital status, loneliness, anxiety and diet. Within this subgroup, less anxious and less lonely males, as well as females with healthier diets, reported better sleep.

**Conclusions:** Sleep quality in older adults was compromised during the sudden unprecedented nation-wide lockdown due to distinct modifiable factors. An important contribution of our study is the assessment of a “clinically-extremely-vulnerable” population and the sex differences identified within this group. Male and female older adults deemed COVID-19CEV may benefit from targeted mental health and dietary interventions, respectively. This work extends the available evidence on the notable impact of lack of social interactions during the COVID-19 pandemic on sleep, and provides recommendations toward areas for future work, including research into vulnerability factors impacting sleep disruption and COVID-19-related complications. Study results may inform tailored interventions targeted at modifiable risk factors to promote optimal sleep; additionally, providing empirical data to support health policy development in this area.

## Introduction

The Coronavirus (COVID-19) pandemic still presents unprecedented global challenges. In the UK, the death toll surpassed, as of April 2021, 125,000 individuals (1). Although the UK vaccination programme is underway (2), the social and economic impacts of the pandemic, as well as the public health measures imposed, are likely to persist beyond the programme's success. Understanding the short- and long-term implications of COVID-19 and government-directed social distancing measures on health, psychosocial and lifestyle outcomes for individuals and society remains imperative. This is important for the population sub-set considered by NHS England to be “clinically extremely vulnerable” to COVID-19-related complications (COVID-19 CEV), due to pre-existing morbidity (3). In the UK, these *at-risk* individuals were encouraged to “shield” during the first lockdown by not leaving their home, except for restricted purposes, such as medical appointments. A large majority of the *at-risk* population are older adults, mostly women (4). However, men overall show higher COVID-related hospital admissions and death (5), indicating sex/gender-differences in risk.

Sleep quality disturbances have been widely studied as key health consequences of the global pandemic, mostly in younger populations and in those subjected to social distancing measures (6). Individual circumstances may vary as a function of age and, to date, there has been relatively little focus on sleep health during lockdowns in older adults (6). Furthermore, there are no published data (to our knowledge) on the impact of sleep in *at-risk* (shielding) groups within a UK sample. This is surprising given that sleep quality correlates with health, psychosocial and cognitive outcomes (7) and may accelerate the development of chronic multimorbidity in older adults (8). Furthermore, shielding may engender greater social isolation, which, as we have previously shown, predicted adverse psychosocial outcomes in older adults during the first UK lockdown (9). Social isolation has been associated with poorer sleep quality in this age-group (10), particularly during the current pandemic (6). Other health and lifestyle factors, such as diet (11) and physical activity (12) may also influence sleep parameters in older adults but have received less attention as possible modifiers in several COVID-19 sleep studies, to date.

The aim of this study is to provide a better understanding of COVID-19 restrictions upon sleep quality and its interaction with other pandemic-affected health and lifestyle outcomes in a UK sample of older adults that include “clinically extremely vulnerable” individuals. Greater knowledge of the pandemic implications for such individuals will inform health policies toward personalised interventions for sustained or enhanced health and well-being during current and future periods of restrictions and isolation, as well as into the recovery phase of the pandemic.

## Methods

### Study Population

Study participants were recruited from the London based Cognitive Health in Ageing Register for Investigational and Observational Trials (CHARIOT; 9) for participation in the CHARIOT COVID-19 Rapid Response (CCRR) Study. The CHARIOT Register was initiated in 2011 through collaborative efforts between GP practises across North and West London and Imperial College London. The register comprises ~40,000 cognitively healthy older adults, aged 50–85 years, at time of recruitment (13).

### Study Design and Assessments

The CCRR study was initiated at Imperial College London, UK, in early April 2020 less than a month after the sudden government-mandated nationwide lockdown. The study aimed to investigate the impact of the COVID-19 pandemic and associated social distancing measures on the mental and physical health of an older adult population. Data were collected via an online repeated survey administered at 6-weekly intervals, before reducing frequency to 3-monthly from September 2020. This cross-sectional investigation reports on the baseline CCRR study data collected during the first COVID-19 pandemic wave in the UK i.e., April–June 2020 (9).

### Demographic, Lifestyle, General, and Mental Health Data

The exploratory variables and covariates included in the study analyses are described in Table 1. In summary, general demographic data included age, sex (collected as “male,” “female,” or “prefer not to say”), marital status, self-isolation, ethnicity and employment status. Data on whether one was shielding and considered clinically extremely vulnerable (COVID-19CEV; 27) was included as primary exposure.

**Table 1:** Population characteristics of study cohort (*n* = 5,558) in relation to COVID-19CEV status.

**Characteristics**	**Total number of participants**	**Covid-19 CEV**	**Non-COVID-19CEV**	***p***
**Age (years) (mean** **±** **SD)**	5,518 (100)	73.1 ± 7.0	70.5 ± 7.3	<0.001
<70 (*n*, %)	2,186 (39.6)	136 (6.22)	2,050 (93.8)	<0.001
≥70 (*n*, %)	3,332 (60.4)	387 (11.6)	2,945 (88.4)	
**Sex** (*n*, %)	5,551 (100)			
Male	2,504 (45.1)	260 (10.4)	2,244 (89.6)	0.026
Female	3,047 (54.9)	263 (8.6)	2,784 (91.4)	
**Marital status (** ***n*** **, %)**	5,554 (100)			
Married/living with partner	3,765 (67.8)	323 (8.6)	3,442 (91.4)	0.002
Single/divorced/widowed	1,789 (32.2)	200 (11.2)	1,589 (88.8)	
**Ethnicity (** ***n*** **, %)**	5,542 (100)			
White	5,214 (94.1)	489 (9.4)	4,725 (90.6)	0.786
Asian/Middle Eastern	164 (3)	19 (11.6)	145 (88.4)	
Black African/Caribbean	35 (0.6)	3 (8.6)	32 (91.4)	
Mixed/other	129 (2.3)	11 (8.5)	118 (91.5)	
**Self-isolating (** ***n*** **, %)**	5,554 (100)			
Yes	640 (11.5)	129 (20.2)	511 (79.8)	<0.001
No	4,914 (88.5)	394 (8)	4,520 (92)	
**BMI (** ***n*** **, %)**	5,558 (100)			
Underweight	44 (0.8)	4 (9.1)	40 (90.9)	0.106
Normal	1,154 (20.8)	100 (8.7)	1,054 (91.3)	
Overweight	671 (12.1)	62 (9.2)	609 (90.8)	
Obese	245 (4.4)	35 (14.3)	210 (85.7)	
**Physical activity (** ***n*** **, %)**	5,558 (100)			
Low	452 (8.1)	52 (11.5)	400 (88.5)	<0.001
Moderate	1,956 (35.2)	172 (8.8)	1,784 (91.2)	
High	2,581 (46.4)	206 (8)	2,375 (92)	
**Employment (** ***n*** **, %)**	5,376 (100)			
Working from home	960 (17.9)	68 (7.1)	892 (92.9)	0.004
Keyworker	184 (3.4)	10 (5.4)	174 (94.6)	
Retired/student	3,932 (73.1)	405 (10.3)	3,527 (89.7)	
Furloughed	300 (5.6)	26 (8.7)	274 (91.3)	
**Lockdown alcohol consumption (** ***n*** **, %)**	4,577 (100)			
Less/same	3,787 (82.7)	336 (8.9)	3,451 (91.1)	0.014
More	790 (17.3)	49 (6.2)	741 (93.8)	
**Smoking status (** ***n*** **, %)**	5,554 (100)			
Yes	178 (3.2)	25 (14)	153 (86)	0.032
No	5,376 (96.8)	498 (9.3)	4,878 (90.7)	
**Lockdown diet (** ***n*** **, %)**	5,554 (100)			
Always healthy	4,443(80)	404 (9.1)	4,039 (90.9)	0.001
Healthy now	619 (11.1)	49 (8)	570 (92)	
Unhealthy now	289 (5.2)	38 (13.1)	251 (86.8)	
Always unhealthy	203 (3.6)	32 (15.8)	171 (84.2)	
**Loneliness (** ***n*** **, %)**	5,547 (100)			
Not ever/rarely	4,038 (72.8)	365 (9)	3,673 (91)	0.204
Sometimes	1,168 (21.1)	116 (9.9)	1,052 (90.1)	
Often	341 (6.1)	40 (11.7)	301 (88.3)	
**Depression (** ***n*** **, %)**	5,548 (100)			
Normal	5,041 (90.9)	447 (8.9)	4,594 (91.1)	<0.001
Borderline	368 (6.6)	55 (15)	313 (85)	
Abnormal	139 (2.5)	20 (14.4)	119 (85.6)	
**Depression score (mean** **±** **SD)**	5,548 (100)	3.86 ± 3.42	3.12 ± 2.85	<0.001
**Anxiety (** ***n*** **, %)**	5,548 (100)			
Normal	4,707 (84.8)	421 (8.9)	4,286 (91.1)	0.004
Borderline	544 (9.8)	58 (10.7)	486 (89.3)	
Abnormal	297 (5.3)	43 (14.5)	254 (85.5)	
**Anxiety score (mean** **±** **SD)**	5,548 (100)	4.66 ± 3.89	4.14 ± 3.35	<0.001
**Chronic comorbidities (** ***n*** **, %)**	5,558 (100)			
2 or less	5,131 (92.3)	425 (8.3)	4,706 (91.7)	<0.001
More than 2	424 (7.7)	98 (23)	329 (77)	
**Sleep quality during lockdown (** ***n*** **, %)**	5,558 (100)			
Good Sleep	3,483 (62.7)	313 (9)	3,170 (91)	0.161
Poor Sleep	2,075 (37.3)	210 (10.1)	1,865 (89.9)	
**Global sleep quality score (mean** **±** **SD)**	5,558 (100)	3.58 ± 2.91	3.28 ± 2.59	0.01

Reported medical comorbidities were computed into a “number of pre-existing conditions” variable, given notable impact on sleep quality (8), and COVID-19 positivity status at time of survey was captured. Height and weight data were self-reported and used to calculate Body mass index (BMI), categorised according to the standard WHO criteria.

**Figure 1:**
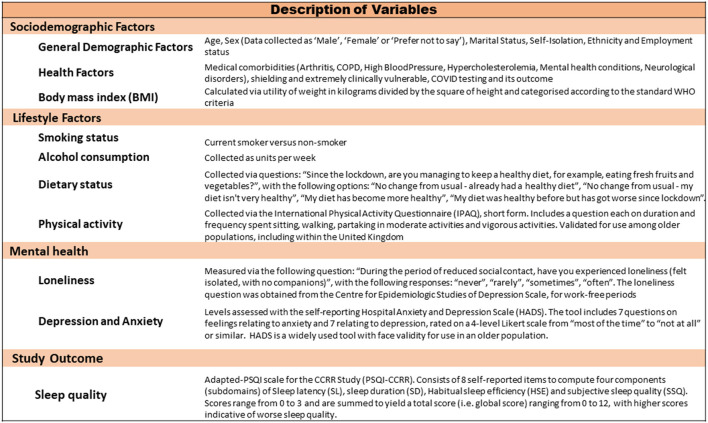
Descriptive summary of study measures.

Lifestyle factors included smoking status, alcohol consumption and diet (described in Figure 1). Physical activity level was collected using the International Physical Activity Questionnaire [IPAQ;(14)].

We assessed self-reported loneliness via the question: “During the period of reduced social contact, have you experienced loneliness (felt isolated, with no companions),” with the following responses: “never,” “rarely,” “sometimes,” “often” [obtained from the Centre for Epidemiologic Studies of Depression Scale, for work-free periods (15)]. Depression and anxiety levels were measured via the Hospital Anxiety and Depression Scale (HADS), described as “Normal,” “Borderline,” or “Abnormal” with higher scale scores indicative of greater anxiety and depressive symptoms (16).

### Sleep Quality (Study Outcome)

The Pittsburgh Sleep Quality Index (PSQI) is one of the most commonly used measures for assessing sleep disturbances in clinical and research settings (17). In the CCRR study, the PSQI was modified to enable assessment of overall sleep quality and individual components before and during the first lockdown period (see Appendix for Supplementary Table 1).

The Adapted-PSQI scale for the CCRR Study (PSQI-CCRR) scale consists of 8 self-reported items to compute four components (subdomains) of sleep latency (SL), sleep duration (SD), habitual sleep efficiency (HSE), and subjective sleep quality (SSQ). Each individual component yields scores ranging from 0 to 3, summed to generate a total score (i.e., global sleep-score) ranging from 0 to 12, with higher scores indicative of worse sleep quality.

### Statistical Analysis

Analyses were performed on a sub-sample of participants with complete sleep data, who undertook baseline CCRR assessments in the period from April to June 2020, corresponding to the first wave of the coronavirus pandemic, peak of case numbers, and fatalities and period of first national lockdown (9).

Sample characteristics are described in relation to global sleep quality and poor sleep. Comparisons of global sleep quality scores with study characteristics were made using analysis of variance (ANOVA) or independent *t*-tests for the continuous measures and chi-squared tests for the categorical variables. Psychometric properties of the Adapted PSQI scale (PSQI-CCRR) were assessed using prescribed tests and indicators (Supplementary Table 2).

Multivariable linear regression analyses adjusting for age, sex and number of pre-existing health conditions were used to independently evaluate associations of global sleep quality during the lockdown with socio-demographic (age, sex, ethnicity, marital/cohabitation status, employment status, COVID-19 positivity and COVID-19CEV status), health/medical (BMI, arthritis, COPD, mental health, neurological conditions, diabetes, high blood pressure and hypercholesterolaemia), lifestyle (alcohol, smoking, diet, and physical activity), psycho-social (loneliness and self-isolating), and mental health (depression and anxiety) factors.

Interactions of COVID-19CEV status with factors significantly associated with sleep quality during lockdown were explored to identify moderators of a hypothesised association of COVID-19CEV status and poor sleep. Sex-stratified analyses of significant moderators of the COVID-19CEV status—sleep quality association were also conducted to investigate potential differences in males and females, and further in relation to sleep quality prior to pandemic-associated lockdown.

All Statistical analyses were performed using STATA (Version 16.1).

## Results

### Population Characteristics in Relation to COVID-19CEV Status and Sleep Quality

Characteristics of the overall CCRR participant population have been previously reported (9). Our analysed sample consisted of CCRR study participants who had completed the sleep quality scale (PSQI-CCRR) from April to June 2020 (Supplementary Table 3: *N* = 5,558). Of these, 55% were females, 94% white, and cohort mean (± SD) age was 71 ± 7.3 years. During lockdown, the mean (± SD) global PSQI-CCRR score was 3.3 ± 2.6, with the 37% participants who scored >3 categorised as poor sleepers. Most participants were married (68%), not self-isolating (88%) and retired (71%). About 8% of participants had more than two chronic diseases and up to 27% reported feeling lonely often or sometimes (Supplementary Table 2).

Overall, 9% of the cohort were CEV (523 out of 5,558). Comparisons between participants deemed high vs. low risk of COVID-19 complications revealed that those in the COVID-19CEV group were more likely to be older, had higher depression and anxiety scale scores, as well as higher global PSQI-CCRR scores, indicative of poorer sleep quality (Table 1). Similarly, the proportion of COVID-19CEV individuals was higher among males, smokers, those who drank less during lockdown and were retired. Significantly higher numbers of COVID-19CEV participants had low lockdown physical activity, suffered from more than two chronic conditions, and reported unhealthy diets during lockdown (Table 1).

No significant differences were noted across ethnicity, BMI or loneliness groups (Table 1).

### Factors Associated With Poor Sleep Quality During COVID-19 Lockdown

Poor sleep quality during lockdown was significantly associated with being in the CEV group (β = 0.35, 95% CI: 0.11–0.58). The association persisted but marginally attenuated, after adjusting for confounders including health and lifestyle determinants of sleep disturbances (β = 0.30, 95% CI: 0.02–0.58) (Table 2). Correspondingly, participants who were not married or co-habiting with a partner as well as those who had higher BMI scores and sufferers of arthritis, COPD or mental health disorders were more likely to report sleeping poorly during the COVID-19 lockdown (see Table 2). Participants who consumed more alcohol (β = 0.35, 95% CI: 0.15–0.55) and had unhealthy diets (β = 0.97, 95% CI: 0.73–1.21) during the early COVID-19 lockdown also reported poorer sleep. Physical activity and smoking status were not significantly associated with sleep quality during the lockdown (Table 2). In relation to psychosocial factors (measures of loneliness and isolation), participants who reported “not ever/rarely” or “sometimes” feeling lonely, as well as those who were not self-isolating were more likely to report sleeping better during the lockdown. On the other hand, anxiety, and depressive symptoms were significantly associated with poorer sleep (β = 0.22, 95% CI: 0.21–0.25 and β = 0.23, 95% CI: 0.21–0.25, respectively). Neither ethnicity, employment status, COVID-19 test positivity nor pre-existing health conditions like diabetes, high blood pressure, asthma, hypercholesterolemia and neurological conditions, were significantly associated with sleep quality (Table 2).

**Table 2:** Factors associated with sleep quality during the early COVID-19 lockdown (April–June 2020).

**Outcome: global sleep quality**		
**Variables**	**β**	**95% CI**
**Covid risk, ref. low #**
**High**	0.35**	[0.11, 0.58]
BMI	0.31*	[0.01, 0.06]
**Self-isolating, ref. no**
Yes	0.25*	[0.03, 0.46]
**Marital status, ref. married/living with partner**
Single/divorced/widowed	0.21**	[0.06, 0.36]
**Alcohol consumption, ref. Less/same**
More	0.35**	[0.15, 0.55]
**Diet, ref. Healthy**
Unhealthy	0.97***	[0.73, 1.21]
**Loneliness code score now, ref. Not Ever/Rarely**
Often	1.72***	[1.44, 2.00]
Sometimes	0.81***	[0.65, 0.98]
**Depression, ref. normal**
Borderline	1.45***	[1.18, 1.72]
Abnormal	2.41***	[1.98, 2.84]
**Anxiety, ref. normal**
Borderline	1.19***	[0.96, 1.42]
Abnormal	2.22***	[1.93, 2.52]
**Depression score**	0.23***	[0.21, 0.25]
**Anxiety score**	0.23***	[0.21, 0.25]

All models Adjusted for sex^***^, age^***^, number of risk factors ^***^; # Model Adjusted for sex^***^, age^***^, number of risk factors ^***^ + Arthritis: Yes (β = 0.36, 95% CI:0.17 to 0.55)^***^, Diabetes: yes (β = 0.14, 95% CI:0.17 to 0.45), Physical Activity: Mets (β = −0.0000127, 95% CI:−0.000364 to 0.00011), High BP (β = 0.12, 95% CI−0.05 to 0.29), Covid-19 positivity status (β = 0.51, 95% CI:−0.57 to 1.59), Hypercholesterolemia (β = 0.03, 95% CI:−0.15 to 0.21), Neurological illness (β = 0.18, 95% CI:−0.58 to 0.95), Mental Health conditions (β = 0.64, 95% CI:0.19 to 1.10)^**^, COPD (β = 0.49, 95% CI:0.07 to 0.92)^*^, Employment status: Unemployed (Retired), ref. Employed (WFH) (β = 0.19, 95% CI:−0.01 to 0.39), Ethnicity: Black African/Caribbean, ref. White (β = 0.58, 95% CI:−0.39 to 1.50), and Smoking: Yes (β = 0.22, 95% CI:−0.61 to 0.17). β refers to unit increases in the outcome and the relation to changes in the investigated predictor variable. Significance at *p <0.05, **p <0.01, ***p <0.001.

### Poor Sleep Quality During Lockdown Among Older Adults at Low and High Risk of Adverse Outcomes Due to COVID-19 (COVID-19CEV Status)

Effect-modification of the positive association between COVID-19CEV status and poorer lockdown sleep quality revealed significant interaction effects for marital status, loneliness, anxiety, and diet (Table 3A). Sub-group analyses in relation to COVID-19CEV status revealed that the previously noted significant positive relationship between good sleep quality with having a healthy diet during lockdown, not ever or rarely feeling lonely and being less anxious during lockdown was more pronounced among COVID-19CEV participants (Diet: Low risk β = −0.82, 95% CI: −1.08, −0.57 and High risk β = −1.77, 95% CI: −2.48, −1.06; Loneliness: Low risk β = −0.74, 95% CI: −1.07, −0.42 and High risk β = −2.14, 95% CI: −3.12, −1.15; Anxiety: Low risk β = −0.22, 95% CI: −0.24, −0.20 and High risk β = −0.30, 95% CI: −0.35, −0.23; All *P* <0.01). The beneficial effect of being married/cohabiting with a partner was only significant among COVID-19CEV participants (β for low risk= −0.15, 95% CI: −0.30, 0.01, *p* = 0.07; β for high risk = −0.78, CI: −1.31, −0.25, *p* = 0.004) (Table 3B).

**Table 3A:** Moderators of the association between COVID-19CEV status and sleep quality during lockdown.

	**Outcome: Global sleep quality**		
**Variables**	**β**	**95% CI**	***P***
Covid risk*Self isolating, ref low
High risk: Self-isolating	–*0.513*	[−1.078, 0.052]	0.075
Covid risk*Marital status, ref. low
High risk: Single/divorced	**0.472**	**[**–**0.006**, –**0.952]**	**0.053**
Covid risk*Lockdown alcohol consumption, ref. low
High risk: More alcohol consumption	0.006	[−0.789, 0.801]	0.988
Covid risk*BMI, ref. low
High risk: BMI	0.042	[−0.033, 0.117]	0.272
Covid risk*Diet
High risk: Unhealthy diet	**0.962**	**[0.267, 1.656]**	**0.007**
Covid risk*Loneliness, ref. low
High risk: Often	**1.539**	**[0.661, 2.417]**	**0.001**
High risk: Sometimes	0.179	[−0.376, 0.736]	0.526
Covid risk*Depression score, ref. low
High risk: Depression score	0.056	[−0.011, 0.123]	0.103
**Covid risk and anxiety score, ref. low**
High risk: Anxiety score	**0.069**	[0.0109, 0.127]	**0.020**

Models Adjusted for sex***, age***, number of risk factors***.

**Table 3B:** Associations of significant moderators of the COVID-19ECV status—sleep quality relationship, among the COVID-19ECV groups.

	**Outcome: global sleep quality**					
	**Covid-19 ECV**			**Non-COVID-19 ECV**		
**Variables**	**β**	**95% CI**	***P***	**β**	**95% CI**	***P***
**Marital status, ref. married/living with partner**
High risk: Single/divorced	0.78	[0.25, 1.31]	0.004	0.15	[−0.11, 0.30]	0.069
**Lockdown diet, ref. healthy diet**
High risk: Unhealthy diet	1.77	[1.65, 2.48]	<0.001	0.82	[0.67, 0.96]	<0.001
**Loneliness, ref. not ever/rarely**
High risk: Sometimes	1.03	[0.45, 1.60]	0.001	0.79	[0.61, 0.96]	<0.001
High risk: Often	3.17	[2.26, 4.08]	<0.001	1.53	[1.23, 1.83]	<0.001
**Anxiety score**	0.29	[0.23, 0.35]	<0.001	0.22	[0.20, 0.24]	<0.001

Models Adjusted for sex***, age***, number of risk factors***.

Modification by depression and self-isolation status was only marginally significant (p-values for interaction: 0.09 and 0.07 respectively). Conversely, the significant positive association between COVID-19CEV status and worsened sleep quality was not moderated by alcohol consumption during lockdown or BMI (p-value for interaction >0.1) (see Table 3A).

### Sex Differences in Sleep Quality During Lockdown Among Older Adults at High vs. Low Risk of COVID-19-Related Complication

In sex-stratified analyses, comparing interaction effect of COVID-19CEV status and modifiable factors (diet, anxiety, marital status and loneliness) with sleep, reporting that “not ever/rarely” feeling lonely and being less anxious were associated with better sleep quality (β = −2.94, 95% CI: −4.56, −1.37), with the interaction term being statistically significant for COVID-19 CEV males only (β = −0.10, 95%CI: −0.19, −0.02). Furthermore, the moderating effect of being married/cohabiting was only marginally significant in males (β = −0.70, 95%CI: −1.45, −0.04; *p* = 0.064). On the contrary, the interaction of healthy diet and COVID-19CEV in relation to better sleep quality was only significant among females (β = −1.16, 95% CI: −2.14, −0.19; Table 4).

**Table 4:** Sex-stratified analysis showing modification of relationship between COVID-19CEV status and sleep quality during lockdown, by distinct lifestyle and psychosocial predictors of sleep, in males and females.

	**Outcome: global sleep quality**					
	**Males**			**Females**		
**Variables**	**β**	**95% CI**	***P***	**β**	**95% CI**	***P***
Covid risk*Marital status, ref. low
High risk: Single/divorced	0.70	[−0.04, 1.45]	0.064	0.50	[−0.18, 1.18]	0.147
Covid risk*Diet						
High risk: Unhealthy diet	0.74	[−0.25, 1.72]	0.142	**1.16**	**[0.19, 2.14]**	**0.020**
Covid risk*Loneliness, ref. low
High risk: Sometimes	0.42	[−0.40, 1.24]	0.312	0.04	[−0.73, 0.81]	0.917
HIGH RISK: Often	**2.94**	**[1.32, 4.56]**	** <0.001**	1.12	[0.03, 2.20]	0.058
Covid risk*Anxiety score, ref. low
High risk: Anxiety score	**0.10**	**[0.02, 0.19]**	**0.020**	0.06	[-0.02, 0.14]	0.173

Models Adjusted for sex***, age***, number of risk factors***.

## Discussion

The last year has witnessed significant global COVID-19 pandemic-related deaths and disabilities. Yet the impact of the pandemic on sleep quality, a significant risk factor for poor cognitive outcomes and gross morbidities in older adults (7, 8) remains largely unknown, particularly for groups at highest risk of medical complications due to COVID-19.

Here we provide the first systematic investigation of sleep quality among UK older adults during the first pandemic wave that featured a complete unparalleled nation-wide lockdown, with a focus on individuals at highest risk of COVID-19-related complications, defined as “clinically extremely vulnerable” or COVID-19CEV (3).

We found that younger participants, females, and those with multimorbidity had poorer sleep quality during lockdown. In addition, sleep quality during the lockdown was poorer among COVID-19 CEV participants, those with higher BMI, those living alone, and among participants suffering from arthritis, COPD or mental health disorders. Of several lifestyle factors investigated, higher alcohol consumption and unhealthy diet were associated with poor sleep during lockdown, whereas no association was observed with smoking and physical activity, during the first lockdown. Of the psychosocial factors, “never or rarely” feeling lonely, and not self-isolating were associated with better sleep quality, whereas higher levels of anxiety and depressive symptoms were associated with poor sleep.

**Figure 2:**
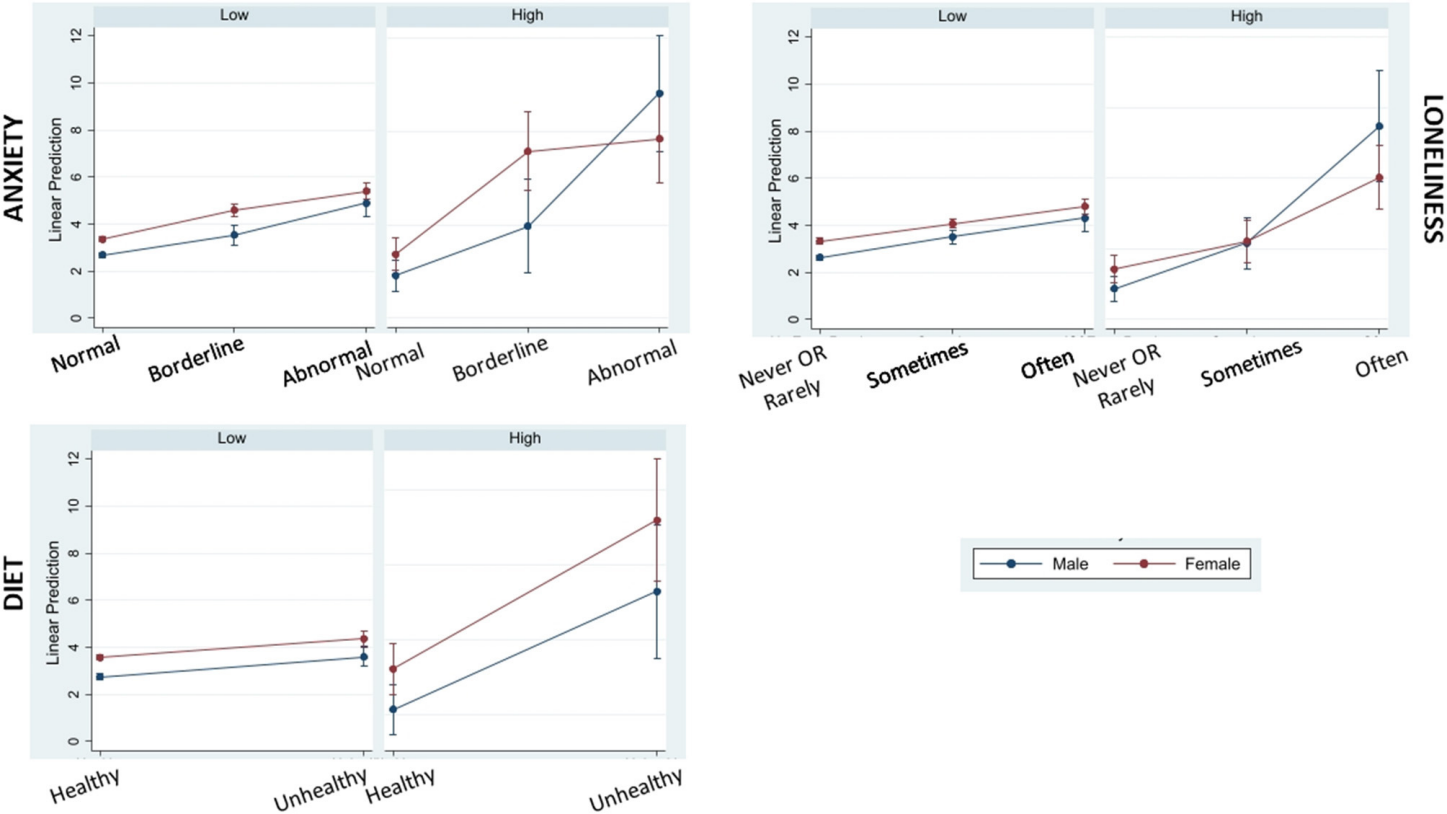
Marginal plots showing interactions of COVID-19 complication risk level (low and high) with significant moderators of poor sleep quality during lockdown (anxiety, loneliness, and diet) by sex (male and female). Higher PSQI-CCRR (sleep scale) scores indicative of poor sleep quality are predicted for males in the Covid High risk category who scored highest in the anxiety component of HADS scale (Abnormal group) and who reported “Often” feeling lonely during the Covid-19 lockdown period. Conversely, higher scores representing poorer sleep quality are predicted for females in the Covid High risk category who had Unhealthy diets during the Covid-19 lockdown period.

Factors that attenuated the notable positive association between COVID-19CEV status and poor sleep quality included being married or cohabiting with a partner, having a healthy diet during lockdown, never or rarely feeling lonely and having lower levels of anxiety symptoms. Older adults with good sleep quality prior to lockdown were most likely to report poor sleep during lockdown if they had unhealthy diets (Supplementary Table 4). Also, pre-lockdown good sleep quality was maintained during the lockdown among married or cohabiting participants (Supplementary Table 4). Sub-group analysis by sex showed that “never” or “rarely” feeling lonely and lower anxiety symptoms were associated with better sleep quality, and that the associations were most pronounced for males with COVID-19CEV status. Among COVID19-CEV females, healthier diet promoted better sleep quality (see Figure 2).

Our study supports previous reports, suggesting the development of sleep disturbances during the pandemic among females, those with chronic illnesses, higher stress levels, higher alcohol consumption, loneliness and depressive symptoms (6, 18). We noted that higher alcohol consumption was associated with poor sleep quality; indeed, high alcohol consumption disrupts sleep architecture and compromises sleep quality, though relationships may be bi-directional (19). Findings surrounding the role of a healthy diet are in line with recent evidence suggesting that healthier dietary patterns (e.g., a Mediterranean diet) are associated with better sleep quality (20). Lifestyle factors, such as alcohol consumption and diet, are arguably more amenable to self-management (thus “more” modifiable), compared with psychosocial factors, such as loneliness and mood, during periods of social restrictions. Therefore, results suggest a potential avenue for intervention to improve sleep quality during lockdowns or periods of self-isolation. Although physical activity can improve sleep quality (21), in the current study, due to lockdown measures, the limited variability in exercise levels may has precluded the assessment of such associations. Our results are also consistent with findings showing that individuals with pre-existing health conditions are more likely to experience sleep disturbances during the lockdown (22). Within the co-morbidities recorded in our study, patients with arthritis, COPD or a mental health disorder were more likely to report poor sleep during lockdown, highlighting possible vulnerabilities for these conditions. Our findings are novel in demonstrating that older adults who are at risk of complications related to COVID-19 are particularly susceptible to having poor sleep. Following the introduction of lockdown measures, good sleep quality was noted in high-risk participants who were married or cohabiting, had healthy diet, felt less lonely or anxious during lockdown (Supplementary Table 4). The protective effects of marriage/ cohabiting were previously observed in a younger sample, albeit without consideration of COVID-complications risk-status (22). Like our study, anxiety and depressive symptoms have been associated with worse sleep quality, potentially related to abnormalities of the hypothalamic-pituitary-adrenal axis and resulting hypercortisolism (23). Unhealthy diets have also been associated with poor sleep quality (11). Such results further highlight the adverse effects of social isolation, loneliness and unhealthy lifestyle with regards to their effects on sleep.

An important contribution of our data is the sex differences observed within the COVID-19CEV group, with sleep quality being influenced more by anxiety and loneliness in males and diet in females. The majority of COVID-19 studies report a disproportional impact of the pandemic on mental health (24) and sleep (6) among women. With respect to high-risk status for COVID-19 complications, our findings highlight that sleep may be more disrupted in CEV males reporting symptoms of anxiety and loneliness, suggesting that specific mental health interventions may be appropriate for this group. On the other hand, females in this high-risk group might benefit from dietary interventions promoting a healthier diet. Similar to our findings, but outside of COVID-lockdown, Jaussent et al. found gender differences in sleep/diet associations, with a Mediterranean diet being protective for sleep quality in women only (25). We did not ask participants to endorse which dietary pattern they followed, but instead whether they believed their diet was “healthy or not during lockdown.” Nonetheless, our results support targeted dietary modification in females at risk from COVID-19 complications. Taken together, these findings underscore the importance of understanding the role of moderating factors, such as sex and clinical vulnerability, in planning strategies for tailored public health intervention or promotion policies.

This study is not without limitations. These include the cross-sectional design (specifically addressing the period of the first lockdown), use of self-reported measures that may be vulnerable to participant bias, lack of data on sleep-related medications, and no objective sleep assessment and/or consensus sleep diary that might more accurately assess sleep quality. However, risk of bias due to cognitive impairment that may precipitate measurement error or data inaccuracies was mitigated as the CCRR study included cognitively healthy older adults from the CHARIOT register (13), thus unlikely to have had significant difficulty in completing the survey accurately. Another key limitation is related to selection bias. Collection of data via on-line surveys may have inadvertently excluded individuals beyond the digital divide. Furthermore, we did not assess coping strategies unlike other studies which found that resilience (i.e., increased adaptability to lockdown challenges) moderated the relationship between social isolation and sleep quality (26). Similarly, we did not record personality trait variables, such as emotional stability, intellect and extraversion, which were associated with improved psychosocial and behavioural outcomes during lockdown in the older old (27). Apart from furlough status, we also did not assess adverse life impacts of the pandemic on sleep quality (28). Furthermore, our sample is overwhelmingly white. Sleep differences have been observed between ethnic groups (29) and a recent report suggested that sleep in ethnic minority groups is disproportionately affected by COVID-19 and lockdown restrictions (30). However, their study considered all age groups over 16 years, did not distinguish those with and without “clinically extremely vulnerable” status and had no data on diet and other lifestyle factors. Future research exploring COVID/lockdown restrictions on sleep quality should aim to explore these factors, and interactions thereof, with ethnicity to better inform targeted public health intervention strategies.

Nonetheless, our study has several strengths. We have used a large sample of older adults, including vulnerable participants at high risk for COVID-19-related complications. Our study is rich in individual-level data, including demographic, lifestyle and psychosocial, as well as measurements of both cohabitant status and feelings of loneliness, and the use of well-validated scales. The retrospective assessment of sleep quality prior to lockdown further allowed evaluation of the specific effect of the pandemic on sleep quality.

In conclusion, the initial UK COVID-19 lockdown had an impact on sleep quality among older adults, with several lifestyle and psychosocial factors playing an important role, that differ by COVID-19CEV status and sex. Importantly, most factors assessed in this study are modifiable and could be targeted to improve sleep among older adults, including those at risk for COVID-19-related complications; through health promotion, psychosocial support, and clinical interventions such as cognitive behavioural therapy for insomnia (CBTi) and sleep hygiene. Within the at-risk group, males and females might benefit from targeted mental health and dietary interventions, respectively. Our findings may inform future interventional research and tailor policy to address poor sleep in older adults and those deemed “clinically extremely vulnerable.” Future work could expand on our research by assessing other vulnerability factors to sleep disruption and COVID-19-related complications, such as ethnicity and polypharmacy. Our next step will examine the longer-term impact of the distinct pandemic waves on sleep quality across distinct domains.

## Data Availability Statement

The raw data supporting the conclusions of this article will be made available by the authors, without undue reservation.

## Ethics Statement

The studies involving human participants were reviewed and approved by data collected as in this study are anonymized and kept strictly confidential in accordance with the UK General Data Protection Regulations (2016). The CCRR study was ethically approved by the Imperial College London Joint Research Compliance Office (20IC5942) and by the Health Research Authority (16/EM/0213). The patients/participants provided their written informed consent to participate in this study.

## Author Contributions

CU-M conceived the sleep project, developed the sleep survey, conducted the analysis, and wrote the draught manuscript, with TW, SS, PG, and CR. SA-A and BZ advised on statistical methods. AW and JM supported with data cleaning and data management activities. CU-M, PG, CR, SA-A, CdJL, DS, TB, GP, CA, AM, AHM, HW, and LM developed the CCRR Study and are Study Investigators, with HW as Principal Investigator. All authors were involved in the interpretation of the results, as well as review and editing of the manuscript.

## Funding

This study was sponsored by Imperial College London and partly funded by the Imperial College Healthcare Trust, NIHR Biomedical Research Centre.

## Conflict of Interest

LM reports clinical trial grants from Janssen R&D, Novartis and Takeda outside the submitted work. The remaining authors declare that the research was conducted in the absence of any commercial or financial relationships that could be construed as a potential conflict of interest.

## Publisher's Note

All claims expressed in this article are solely those of the authors and do not necessarily represent those of their affiliated organizations, or those of the publisher, the editors and the reviewers. Any product that may be evaluated in this article, or claim that may be made by its manufacturer, is not guaranteed or endorsed by the publisher.
