# Supplementary material for: Health, Lifestyle, and Psycho-Social Determinants of Poor Sleep Quality During the Early Phase of the COVID-19 Pandemic: A Focus on UK Older Adults Deemed Clinically Extremely Vulnerable
Source: Front Public Health. 2021 Oct 28;9:753964. doi: 10.3389/fpubh.2021.753964 (PMC8637825; doi:10.3389/fpubh.2021.753964)
Supplement: Supplementary file 1 [file Data_Sheet_1.PDF]

Supplemental Information

Title: Health, lifestyle and psycho-social determinants of poor sleep quality during the Early Phase of the COVID-19 pandemic: a focus on UK older adults deemed clinically extremely vulnerable

Running Title: Sleep Quality in at-risk older adults during the COVID-19 pandemic

Chinedu T Udeh-Momoh[1*], Tamlyn Watermeyer[2,3], Shireen Sindi[1,4], Parthenia Giannakopoulou[1], Catherine E Robb[1], Sara Ahmadi-Abhari[1], Bang Zheng[1], Amina Waheed[1], James McKeand[1], David Salman[5,6], Thomas Beaney[6], Celeste A. de Jager Loots[1], Geraint Price[1], Christina Atchison[7,11], Josip Car,[6,7,8], Azeem Majeed,[6,7], Alison. H. McGregor[9], Miia Kivipelto[1,4,10,11], Helen Ward[7,12] and Lefkos T. Middleton[1,7]

Author affiliations:

[1]Ageing Epidemiology Research Unit (AGE), School of Public Health, Faculty of Medicine, Imperial College London, UK

[2]Department of Psychology, Faculty of Health and Life Sciences, Northumbria University, Newcastle, UK

[3] Edinburgh Dementia Prevention, Centre for Clinical Brain Sciences, University of Edinburgh, Edinburgh, UK

[4] Division of Clinical Geriatrics, Center for Alzheimer Research, Karolinska Institutet and Karolinska University Hospital, Stockholm, Sweden

[5]MSk lab, Faculty of Medicine, Imperial College London, UK

[6]Department of Primary Care and Public Health, Imperial College London, UK

[7]Public Health Directorate, Imperial College Healthcare NHS Trust, London, UK

[8]Centre for Population Health Sciences, Lee Kong Chian School of Medicine, Nanyang Technological University, Singapore.

[9]Department of Surgery and Cancer, Imperial College London, UK

[10]Theme Aging, Karolinska University Hospital, Stockholm, Sweden

[11]Institute of Public Health and Clinical Nutrition and Institute of Clinical Medicine, Neurology, University of Eastern Finland, Kuopio, Finland

[12]Patient Experience Research Centre, School of Public Health, Faculty of Medicine, Imperial College London, UK

Appendix 1a: The PSQI-CCRR scale

Supplemental Table 1: Questions and Scoring guide for the adapted PSQI-CCRR Scale

Scale Questions	Scoring Instructions
**The PSQI-CCRR Questionnaire**<br>The questionnaire is adapted from the Pittsburgh Sleep Quality Index -PSQI (Buyse et al 1989) and Center for Epidemiologic Studies of Depression Scale -CES-D.<br>The 7-item scale assesses the impact of social isolation on components of sleep latency, duration, efficiency and subjective sleep quality. This self-report questionnaire examines sleep habits before and during a period of decreased social interactions, for a period of 1 month.<br>**Instructions:**<br>The following questions relate to your usual sleep habits **for a period of one month** before and during a period of reduced social contact (RSC). Your answers should indicate the most accurate reply for the majority of days and nights during these periods.<br>Please answer all questions.<br>**1.** During the period before reduced social contact, what time have you usually gone to bed at night: before RSC bed-time is (e.g. 10pm = 22:00) - **24-hour clock**<br>1b) During the period of reduced social contact, what time have you usually gone to bed at night: RSC bed-time is - **24-hour clock**<br>**2.** During the period before reduced social contact, how long (in minutes) did it usually take you to fall asleep each night: before RSC number of minutes<br>2b) During the period of reduced social contact, how long (in minutes) has it usually taken you to fall asleep each night: RSC number of minutes<br>3. During the period before reduced social contact, what time have you usually gotten up in the morning: before RSC getting-up time is - **24-hour clock**	Each component score will be calculated for before RSC and during RSC.<br>Total sleep quality will be computed as a sum of all components.<br>Increasing values indicate poorer sleep quality.<br>**Sleep Latency score (from Q2 and 5):**<br>Sleep latency sub-score a (Response to Q2): < 15 minutes: 0; 16-30 minutes: 1; 31-60 minutes: 2; > 60 minutes: 3<br>Sleep latency sub-score b (Response to Q5): Not during past month: 0; Less than once a week: 1; Once or twice a week: 2; Three or more times a week: 3<br>Sleep latency score = Sum of sub-scores a and b: 1-2: **1**; 3-4: **2**; 5-6: **3**<br>**Sleep Duration score (from Q4):**<br>Sleep duration (Response to Q4): > 7 hours: 0; 6-7 hours: 1; 5 hours: 2; < 5 hours: 3<br>**Sleep Efficiency score (from Q1,3,4):**<br>Calculate<br>Sleep efficiency sub-score a: total no hours slept (from Q4)<br>Sleep efficiency sub-score b: total no hours in bed (Subtract Q3 from Q1 i.e. wake up time from bed time)<br>Compute (a/b)*100<br>Sleep efficiency total score: > 85%: 0; 75-84%: 1; 65-74%: 2; < 65%: 3<br>**Subjective sleep quality (Question 6):**

3b) During the period of reduced social contact, what time have you usually gotten up in the morning: RSC getting-up time is – [**24-hour clock]**<br>4. During the period before reduced social contact, how many hours of actual sleep did you get at night? (This may be different from the number of hours you spent in bed): before RSC hours of sleep per night is - **(X hours)**<br>4b) During the period of reduced social contact, how many hours of actual sleep do you get at night? (This may be different from the number of hours you spend in bed): RSC hours of sleep per night is - **(X hours)**<br>5. During the period before reduced social contact, how often have you had trouble sleeping because you could not get to sleep within 30 minutes<br>5b) During the period of reduced social contact, how often have you had trouble sleeping because you could not get to<br>sleep within 30 minutes<br>6. During the period before reduced social contact, have you experienced poor sleep (restless and unable to sleep)<br>6b) During the period of reduced social contact, have you experienced poor sleep (restless and unable to sleep)<br>**Responses for Questions 5,5b,6 and 6b are:**<br>o Not ever<br>o Less than once a week<br>o Once or twice a week<br>o Three or more times a week	Not ever: 0; Less than once a week: 1; Once or twice a week: 2; Three or more times a week: 3<br>**Loneliness (**see main text):<br>response options range from 0 to 2 (0 = Rarely or Not ever, 1 = Sometimes or, 2 = Often). High score is indicative of greater symptoms. This score was added to directly assess association of loneliness and sleep quality to test the hypothesis that individuals who report feelings of loneliness due to reduced social contact are most likely to experience poor sleep.<br>**Total sleep score = Subjective sleep quality + Sleep Efficiency score + Sleep Duration score + Sleep Latency score (range = 0-12) higher scores are indicative of poorer sleep.**

Appendix 2: Psychometric Validation of the PSQI-CCRR scale

Multiple psychometric properties of the Adapted PSQI scale (PSQI-CCRR) were analysed using relevant tests and indicators. Specifically, internal consistency reliability of the items and components was assessed using Cronbach's α coefficients and half-split reliability coefficients. Correlations between global and sub-component PSQI-CCRR scores and the results of HADS scale scores for depression and anxiety were examined to assess the convergent validity. Known-groups discriminative validity was evaluated using independent sample t-test and analysis of variance (ANOVA) with Bonferroni correction to test for differences of global sleep quality score across sex groups (male and female), and 'feeling of loneliness during lockdown' responses (classified as Not Ever/Rarely, Sometimes or Often) respectively. Paired t-tests were further used to evaluate differences in sleep quality before and during lockdown, for global sleep and component scores.

Assessment of the reliability and validity of the Adapted PSQI Scale (PSQI-CCRR)

Empirical assessment of the Adapted PSQI questionnaire was performed to test scale validity and reliability of the responses for measuring sleep quality. To assess internal consistency reliability, the Cronbach's α coefficient and half-split reliability tests were conducted. The Cronbach's α coefficient of the four component scores was **0.719 and 0.742** (for sleep quality before and during lockdown respectively), and the corresponding half-split reliability coefficient was **0.768 and 0.792** (for sleep quality before and during lockdown respectively), indicating adequate internal consistency reliability at component level.

For convergent validity, correlations between global PSQI-CCRR scores as well as sub-components of the sleep scale, and the HADS Depression and Anxiety sub-scales were examined. PSQI-CCRR global score was positively correlated with HADS depression and anxiety component scores ($r=0.276$, $p=0.01$, and $r=0.321$, $p=0.01$, respectively), indicative of increased presence of anxiety and depressive symptoms for participants reporting worse sleep quality (Supplemental Table 1a). More so, all four components of PSQI-CCRR were positively correlated with HADS depression and anxiety scales scores (All at $p<0.05$), indicative of good convergent validity (Supplemental Table 2a). Known-groups discriminative validity was further tested by evaluating between-group differences in PSQI global scores across sex and loneliness groups. PSQI-CCRR global score differed by sex and loneliness ($p<0.0001$), such that male participants and those who reported 'rarely or not ever' feeling lonely during lockdown had lower sleep quality scores, indicative of better sleep quality and good discriminative validity (Supplemental Table 2b). Additionally, the components of 'Sleep Latency', and 'Sleep Efficiency' also showed good discriminative validity across sex and loneliness sub-groups, though Sleep duration did not discriminate between sex groups as well as loneliness groups (Supplemental Table 2b).

Further examination of sleep quality before and during the lockdown revealed significant differences for global sleep, sleep latency, efficiency and subjective sleep (All at $p<0.001$). Differences were only marginally significant for sleep duration ($p=0.079$), though higher scores were also noted during lockdown suggestive of worsened sleep duration at that timepoint (Supplemental Table 2c).

Supplemental Table 2a: Assessment of Convergent validity of the PSQI-CCRR Scale

HADS Subscales	ICSQ Global score	Subjective sleep quality	Sleep latency	Sleep duration	Sleep efficiency
**Depression**	**0.276****	0.296**	0.255*	0.134**	0.131**
**Anxiety**	**0.321****	0.390**	0.299*	0.147**	0.149**

Supplemental Table 2b: Assessment of Discriminative validity of the PSQI-CCRR Scale

	**Sex***		**Loneliness#**		
Scale	Male	Female	Rarely/Not Ever	Sometimes	Often
**Global ICSQ**	2.86 ± 3.47	3.68 ± 2.69	2.99 ± 2.46	3.94 ± 2.73	4.92 ± 3.13
P-value	**0.0001**		**0.0001**		

Supplemental Table 2c: Differences in Global and Sub-domains of Sleep Quality before and during the COVID-19 lockdown

	**Paired Differences**			
**Predictors**	**Mean**	**SD**	**95% (CI)**	***P- value***
Total Sleep Quality Score	-0.266	1.572	[-0.308, -0.225]	**0.001**
Sleep Duration	-0.014	0.504	[-0.029, 0.002]	*0.079*
Sleep Latency Total Category Score	-0.047	0.534	[-0.064, -0.031]	**0.001**
Sleep Efficiency Score	1.718	12.063	[0.187, 1.350]	**0.001**
Subjective Sleep Quality	-0.098	0.654	[-0.115, -0.81]	**0.001**

Note: difference below 0 means worse sleep quality during lockdown.

Appendix 3: Analysis of the PSQI-CCRR scale

Supplemental Table 3: Population characteristics in relation to Sleep Quality

Characteristics	Number of participants	Global Sleep		Global Sleep > 3	
		$\bar{x}$ ± SD	*P* value	n (%)	*P* value
**Total**	5558	3.3± 2.6		2075 (37.3)	
**Age categories (n, %)**	5518 (100)		0.003		<0.001
<70	2186 (39.6)	3.59 ± 2.66		927 (42.4)	
≥70	3332 (60.4)	3.11 ± 2.58		1135 (34.1)	
**Sex (n, %)**	5551 (100)		0.001		<0.001
Male	2504 (45.1)	2.86 ± 2.47		776 (31.0)	
Female	3047 (54.8)	3.68 ± 2.69		1299 (42.6)	
**Marital status (n, %)**	5554 (100)		0.001		0.006
Married/Living with Partner	3765 (67.7)	3.19 ± 2.55		1360 (36.12)	
Single/Divorced/Widowed	1789(32.2)	3.55 ± 2.75		715 (39.97)	
**Ethnicity (n, %)**	5542 (100)		0.326		0.038
White	5214 (93.8)	3.30 ± 2.62		1935 (37.11)	
Asian/Middle Eastern	164 (3)	3.23 ± 2.73		63 (38.4)	
Black African/Caribbean	35 (0.6)	4.09 ± 2.41		21 (60.0)	
Mixed/Other	129 (2.3)	3.42 ± 2.70		53 (41.2)	
**Self-Isolating (n, %)**	5554 (100)		0.38		0.409
Yes	640 (11.5)	3.41 ± 2.69		249 (38.9)	
No	4914 (88.4)	3.29 ± 2.61		1826 (37.16)	
**BMI (n, %)**	5558 (100)		0.001		<0.001
Underweight (<18.5)	44 (0.8)	4.05 ± 2.53		28 (63.64)	
Normal (18.5 – 24.9)	1154 (20.8)	3.89 ± 2.62		561 (48.61)	
Overweight (25.0 – 29.9)	671 (12.1)	3.98 ± 2.75		331 (49.33)	
Obese (≥ 30.0)	245 (4.4)	4.09 ± 2.57		133 (91.5)	
**Physical Activity (n, %)**	5558 (100)		0.33		0.378

Low	452 (8.1)	3.41 ± 2.73		180 (39.82)	
Moderate	1956 (35.2)	2.26 ± 2.56		706 (36.09)	
High	2581 (46.4)	2.28 ± 2.58		948 (37.50)	
**Employment (n, %)**	5376 (100)		0.167		0.047
Working from Home	960 (17.3)	3.24 ± 2.50		354 (36.88)	
Keyworker	184 (3.3)	3.49 ± 2.72		77 (41.85)	
Retired/Student	3932 (70.7)	3.27 ± 2.63		1434 (36.47)	
Furloughed	300 (5.4)	3.57 ± 2.65		131 (43.67)	
**Alcohol Consumption (n, %)**	4577 (100)		0.54		0.001
Less/Same in lockdown	3787 (68.1)	3.20 ± 3.58		1354 (35.75)	
More in lockdown	790 (14.2)	3.64 ± 2.67		332 (42.03)	
**Smoking (n, %)**	5554 (100)		0.46		1.000
Yes	178 (3.20)	3.16 ± 2.64		66 (37.08)	
No	5376 (96.8)	3.31 ± 2.62		2009 (37.37)	
**Diet (n, %)**	5554 (100)		0.001		<0.001
Always Healthy	4443(80)	3.19 ± 2.57		1592 (35.83)	
Healthy Now	619 (11.1)	3.21 ± 2.56		239 (38.61)	
Unhealthy Now	289 (5.2)	4.32 ± 2.99		154 (53.29)	
Always Unhealthy	203 (3.65)	4.03 ± 3.02		90 (44.33)	
**Loneliness (n, %)**	5547 (100)		0.001		<0.001
Not ever/ Rarely	4038 (72.8)	2.99 ± 2.45		1339 (33.16)	
Sometimes	1168 (21.1)	3.94 ± 2.73		547 (46.83)	
Often	341 (6.1)	4.92 ± 3.13		189 (55.43)	
**Depression (n, %)**	5548 (100)		0.001		<0.001
Normal	5041 (90.7)	3.14 ± 2.50		1779 (35.3)	
Borderline	368 (6.6)	4.74 ± 3.01		204 (55.43)	
Abnormal	139 (2.5)	5.80 ± 3.33		92 (52)	
**Anxiety (n, %)**	5548 (100)		0.001		<0.001
Normal	4707 (84.7)	3.05 ± 2.47		1598 (33.95)	

Borderline	544 (9.8)	4.39 ± 2.78		282 (51.84)	
Abnormal	297 (5.3)	5.49 ± 3.04		195 (65.66)	
**Chronic Comorbidities (n, %)**	5558 (100)		0.003		0.009
2 or less	5131 (92.3)	3.26 ± 2.60		1890 (36.83)	
More than 2	424 (7.7)	3.85 ± 2.83		185 (43.33)	
**COVID19CEV Status (n, %)**	5558 (100)		0.01		0.168
No	5035 (90.6)	3.28 ± 2.59		1865 (37.04)	
Yes	523 (9.4)	3.58 ± 2.91		210 (40.15)	

Appendix 4: Sex differences in the association of sleep quality during lockdown, with lifestyle and psycho-social factors, in relation to sleep quality before the lockdown, and among older adults at high versus low risk of COVID-19

We evaluated associations between sleep quality during lockdown with lifestyle and psychosocial predictors of sleep, in relation to COVID-19CEV status, pre-lockdown sleep quality (Supplemental Table 4).

Participants who had good sleep quality before lockdown and whose diet changed to an unhealthy one during the lockdown period had poorer sleep during lockdown, alluding to a moderating role of nutrition for better sleep quality in those at highest risk of the adverse effects of COVID-19. Irrespective of sleep quality before or during the lockdown, participants who were not ever lonely and less anxious or depressed during lockdown, all reported better sleep quality during the lockdown period, though the effects were most notable in the COVID-high risk participant group. Similarly, those who had good sleep quality before the lockdown and were married or cohabiting with a partner also had good sleep during lockdown.

Supplemental Table 4: Associations between sleep quality during lockdown with lifestyle and psychosocial predictors of sleep, in relation to COVID-19CEV status and pre-lockdown sleep quality.

Predictors	Low Covid Risk, β [95% CI]		High Covid Risk β [95% CI]	
	Good Sleep	Poor Sleep	Good Sleep	Poor Sleep
**Diet since Lockdown, ref. always healthy**				
Healthy during lockdown	0.158[-0.023, 0.340]	-0.190 [-0.537, 0.156]	-0.150 [-0.798, 0.498]	0.450 [-0.760, 1.662]
Unhealthy during lockdown	**0.882[0.605, 1.159] *** **	**0.566 [0.099, 1.032] ** **	**1.593 [0.808, 2.379] *** **	**1.987 [0.749, 3.226] *** **
Always Unhealthy	0.198 [-0.130, 0.527]	**0.806 [0.226, 1.386] *** **	-0.121 [-0.978, 0.734]	1.687[0.371, 3.003] **
**Marital Status, ref. Married/Living with partner**				
Single/Divorced/Widowed	**0.147 [0.019, 0.276] * **	0.232 [-0.010, 0.476]	**0.500 [0.081, 0.919] ** **	0.442 [-0.323, 1.207]
**Loneliness code score now, ref. Not Ever/Rarely**				
Often	**0.936 [0.677, -1.196] *** **	1.570 [1.157, 1.984] ***	2.753 [1.951, 3.555] ***	**2.058 [0.919, 3.198] *** **
Sometimes	**0.608 [0.464, 0.753] *** **	**0.657 [0.395, 0.919] *** **	0.428 [-0.018, 0.857]	**0.810 [-0.019, 1.641] * **
**Anxiety Category, ref. normal**				
Borderline	**0.897 [0.696, 1.098] *** **	**0.812 [0.472, 1.152] *** **	0.287 [-0.360, 0.934]	**1.465 [0.471, 2.458] ** **
Abnormal	**1.508 [1.212, 1.803] *** **	**1.549 [1.135, 1.964] *** **	**2.740 [1.860, 3.620] *** **	**1.243 [0.210, 2.277] ** **
**Anxiety score**	**0.162 [0.144, 0.179] *** **	**0.149 [0.118, 0.180] *** **	**0.186 [0.132, 0.239] *** **	**0.183 [0.101, 0.266] *** **

Note: All Models Adjusted for sex***, age***, number of risk factors ***, *Significance at p<0.001***, p<0.01**, p<0.05**
